# A pilot study on implementing a pharmacist-led holistic smoking cessation clinic in a psychiatric hospital

**DOI:** 10.1007/s44192-026-00489-2

**Published:** 2026-05-31

**Authors:** Tirapada Kiatsangorn, Nitikorn Phoosuwan, Sakarin Kaewhao, Somkid Somsiri, Ekasak Raksachat

**Affiliations:** 1 Nakhon Phanom Rajanagarindra Psychiatric Hospital, Nakhon Phanom, Thailand; 2 Faculty of Public Health, Thammasat University, Khlong Luang, Pathum Thani Thailand; 3 Department of Public Health and Caring Sciences, Uppsala University, BMC, Husargatan 3, Box 564, Uppsala, Sweden; 4 Department of Mental Health, Bureau of Mental Health Academic Affairs, Ministry of Public Health, Nonthaburi, Thailand

**Keywords:** Pharmacist-led intervention, Smoking cessation, Psychiatric hospital, Implementation science, Pilot study, Mental health services

## Abstract

**Background:**

Smoking prevalence remains disproportionately high among individuals with psychiatric disorders. In Thailand, structured smoking cessation services are rarely embedded within routine psychiatric care. This pilot study describes the development and early evaluation of a pharmacist-led holistic smoking cessation clinic integrated into a tertiary psychiatric hospital in Northeastern Thailand.

**Methods:**

This hospital-based service development project employed a two-phase design. Phase 1 involved stakeholder-informed co-design of the service model. Phase 2 consisted of pilot implementation among psychiatric outpatients with stable mental health status. During the recruitment period, 22 patients were screened and 18 were enrolled (intention-to-treat sample). The intervention combined pharmacologic support—including nicotine gum (2 mg), cytisine (1.5 mg titration over 25 days when clinically appropriate), and *Cyanthillium cinereum* (formerly *Vernonia cinerea*) herbal tea—with behavioral counseling and multidisciplinary follow-up over 12 weeks. Primary outcomes included feasibility and acceptability; secondary outcomes assessed preliminary smoking-related and psychological indicators.

**Results:**

Of the 18 enrolled participants, 15 completed the 12-week program. Recruitment (81.8%), retention (83.3%), and session adherence (88.9%) exceeded predefined feasibility thresholds. At Week 12, 7-day point prevalence abstinence was 44.4% (8/18, intention-to-treat), and daily cigarette consumption significantly decreased. Patient satisfaction scores were high (mean 4.47/5). No serious adverse events or psychiatric relapses were observed.

**Conclusion:**

This pilot service demonstrates that a pharmacist-led holistic smoking cessation model can be feasibly integrated into psychiatric care in a provincial Thai hospital. Further controlled studies are warranted to evaluate long-term effectiveness and scalability.

## Introduction

Tobacco use remains one of the most significant preventable causes of morbidity and mortality globally, with individuals diagnosed with psychiatric disorders experiencing a disproportionate burden. Evidence shows that people with mental health conditions are two to three times more likely to smoke than the general population, often exhibiting greater nicotine dependence and lower cessation success rates [10, 13]. Among psychiatric patients, tobacco use not only aggravates physical comorbidities—such as cardiovascular and respiratory diseases—but also interferes with psychotropic medications. Cigarette smoke induces hepatic enzymes (e.g., CYP1A2), potentially altering plasma concentrations and therapeutic response of certain antipsychotics and antidepressants [9].

Despite these well-documented risks, smoking cessation services remain underutilized or entirely absent in many mental health care settings, particularly within low- and middle-income countries (LMICs) (World Health Organization, [16]). A key contributor to this service gap is the lack of integration between tobacco cessation and psychiatric care. Conventional referral-based models, which direct psychiatric patients to external cessation programs, have shown limited effectiveness. In contrast, integrated care models that embed smoking cessation into routine psychiatric services demonstrate improved quit rates and better patient outcomes [2].

Pharmacists have increasingly become critical contributors in smoking cessation interventions due to their accessibility, medication management expertise, and expanding roles in multidisciplinary teams. Pharmacist-led models, particularly when combining pharmacotherapy with behavioral support, are associated with improved cessation outcomes [11, 14]. However, in Southeast Asia—including Thailand—such pharmacist-driven models remain underdeveloped in psychiatric hospital settings. In addition, smoking cessation services may also incorporate locally available pharmacologic and complementary approaches to enhance patient engagement.

Nakhon Phanom Rajanagarindra Psychiatric Hospital, a tertiary-level mental health facility in Northeastern Thailand, serves a broad range of psychiatric populations through its inpatient, outpatient, and specialty clinics [7] . Prior to this initiative, there was no structured pharmacist-led smoking cessation service available. Guided by principles from implementation science frameworks [8], the hospital initiated a pilot smoking cessation service outside regular clinic hours, led by a psychiatric pharmacist in collaboration with psychiatrists and mental health professionals. The goal of this initiative was to develop and evaluate the feasibility of integrating a pharmacist-led holistic smoking cessation model into psychiatric care tailored to the complex and contextualized needs of patients in a real-world hospital setting.

## Aim

This pilot service development study aimed to design, implement, and evaluate the feasibility and acceptability of a pharmacist-led holistic smoking cessation clinic embedded within routine psychiatric care at Nakhon Phanom Rajanagarindra Psychiatric Hospital.

Objectives

1. To develop and operationalize a pharmacist-led holistic smoking cessation model tailored to psychiatric outpatient services.
2. To evaluate the feasibility and acceptability of implementing the model in a real-world tertiary psychiatric hospital setting.
3. To assess preliminary clinical and patient-reported outcomes, including smoking behavior change and psychological quality of life, following integration of the model into routine care.

## Method

### Study design

This pilot initiative employed a mixed-methods exploratory sequential design comprising two stages: (1) qualitative exploration and co-design of the service model, and (2) pilot implementation with formative evaluation of a pharmacist-led holistic smoking cessation clinic embedded in psychiatric care. Findings from Stage 1 informed the structure, workflow, and intervention components applied in Stage 2.

The study was conducted as a hospital-based service development initiative aimed at enhancing the quality and accessibility of smoking cessation support within routine psychiatric services.

### Stage 1: qualitative exploration and model development

#### Participants and setting

Key stakeholders were purposefully selected to represent diverse perspectives relevant to service co-design. Participants included psychiatrists (*n* = 3), clinical pharmacists (*n* = 3), psychiatric nurses (*n* = 5), patients with psychiatric disorders who smoke (*n* = 12), and family caregivers (*n* = 6). Healthcare professionals were invited based on their direct involvement in psychiatric outpatient services. Patients were eligible if they were adult outpatients with a history of smoking and clinically stable (i.e., no acute psychiatric episode) at the time of recruitment. Family caregivers were invited if they were actively involved in supporting a psychiatric patient receiving care at the hospital. All participants were recruited from inpatient and outpatient units at Nakhon Phanom Rajanagarindra Psychiatric Hospital.

#### Data collection

Semi-structured interviews and focus group discussions were conducted using topic guides developed by the hospital-based research team. Interviews were facilitated by a trained member of the research team and lasted approximately 45–60 min. With participants’ permission, discussions were audio-recorded and supplemented by field notes.

Discussion topics included perceived barriers to smoking cessation, motivational factors, prior quit attempts, and expectations regarding pharmacist involvement in cessation care. Field observations and review of institutional documents complemented the interviews to assess organizational readiness and contextual factors relevant to service integration.

Example questions included: “What challenges have you faced when trying to quit smoking?”, “What kind of support would help psychiatric patients stop smoking?”, and “How do you perceive the role of pharmacists in smoking cessation care?”

#### Data analysis

Interview transcripts were analyzed inductively using Braun and Clarke’s six-phase framework for thematic analysis [1]. Two researchers independently coded the transcripts and subsequently met to compare interpretations and resolve discrepancies through discussion. Peer debriefing was conducted to enhance analytical credibility. The final themes were used to refine the structure, workflow, and intervention components of the pilot clinic.

### Stage 2: pilot implementation and formative evaluation

#### Participants

During the recruitment period (January–May 2025), 22 psychiatric outpatients were screened, whereas 18 met inclusion criteria and were enrolled in the pilot clinic (intention-to-treat sample).

Eligibility criteria were people: aged 18–65 years; daily smoked of ≥ 10 cigarettes for ≥ one year; had no acute psychotic episode within the previous month; and had capacity to provide informed consent. The ≥ 10 cigarettes/day threshold was used to ensure inclusion of regular daily smokers with established nicotine exposure. Nicotine dependence level was further assessed using the Fagerström Test for Nicotine Dependence (FTND).

Exclusion criteria included diagnosed cognitive impairment or dementia, pregnancy or breastfeeding, and known hypersensitivity to nicotine replacement therapy (NRT) or cytisine.

Participants were identified during routine outpatient psychiatric appointments and invited to participate by the treating psychiatrist or pharmacist.

#### Service model

The pilot clinic was delivered by a psychiatric pharmacist in collaboration with psychiatrists and psychiatric nurses. All clinic consultations were conducted as individual in-person sessions and were provided free of charge as part of routine hospital services. Prior to implementation, the pharmacist completed focused training in motivational interviewing and smoking cessation counseling.

The holistic care model integrated pharmacologic treatment, behavioral support, and psychoeducation, comprising the following components:

- Baseline assessment: Fagerström Test for Nicotine Dependence (FTND), exhaled carbon monoxide (CO) measurement, and readiness-to-quit assessment.
- Individualized treatment planning based on nicotine dependence level, patient preference, and psychiatric stability.
- Pharmacologic options:
  - Nicotine gum (2 mg), administered every 1–2 h as needed (maximum 15 pieces per day).
  - Cytisine (1.5 mg), prescribed using a standard 25-day tapering regimen for participants with moderate-to-high nicotine dependence and no contraindications.
  - *Cyanthillium cinereum* (formerly *Vernonia cinerea*) herbal tea, provided as adjunctive support in a standardized preparation (twice daily).
- Scheduled follow-up visits at Weeks 1, 2, 4, 8, and 12.
- Family engagement and reinforcement of social support strategies.
- Weekly interdisciplinary case discussions for medication review and monitoring of psychiatric stability.

#### Outcome measures

The primary outcomes were feasibility and acceptability. Feasibility was defined as achieving ≥ 80% recruitment, retention, and follow-up completion among enrolled participants. Acceptability was defined as a mean patient satisfaction score ≥ 4.0 on a 5-point Likert scale. Patient satisfaction was assessed using a brief structured questionnaire evaluating accessibility of the clinic, clarity of counseling, perceived helpfulness of the service, and overall service experience.

Secondary outcomes included:

- 7-day and 30-day point prevalence abstinence (self-report verified by exhaled CO ≤ 6 ppm),
- Change in daily cigarette consumption,
- Change in motivation to quit (Motivation to Quit Scale, Thai version), and.
- Change in psychological quality of life (WHOQOL-BREF, Thai version).

#### Data analysis

Quantitative data were analyzed using descriptive statistics and paired t-tests for pre–post comparisons. Effect sizes were calculated using Hedges’ g to adjust for small sample bias. 95% confidence intervals were reported where appropriate.

An intention-to-treat (ITT) approach was applied, with missing outcome data handled using the last observation carried forward (LOCF) method to provide conservative estimates.

## Results

A total of 18 participants were included in the intention-to-treat analysis. Baseline characteristics of the enrolled participants are presented in Table 1.

**Table 1:** Baseline characteristics of participants (*N* = 18)

Characteristic	Value
Age, mean (SD)	39.6 (10.8) years
Gender, n (%)	
Male	14 (77.8)
Female	4 (22.2)
Primary psychiatric diagnosis, n (%)	
Schizophrenia (F20.x)	10 (55.6)
Bipolar disorder (F31.x)	5 (27.8)
Schizoaffective disorder (F25.x)	2 (11.1)
Major depressive disorder	1 (5.5)
Duration of illness, n (%)	
≤5 years	5 (27.8)
6–10 years	6 (33.3)
>10 years	7 (38.9)
Daily cigarette consumption, mean (SD)	18.3 (4.2)
Smoking duration (years), mean (SD)	13.5 (6.1)
Nicotine dependence (FTND), n (%)	
Low (0–3)	2 (11.1)
Moderate (4–6)	9 (50.0)
High (7–10)	7 (38.9)
Previous quit attempts, n (%)	
None	6 (33.3)
1–2 times	8 (44.4)
≥3 times	4 (22.2)
Co-morbid substance use, n (%)	5 (27.8)
Stable psychiatric medication regimen, n (%)	18 (100)

SD = Standard Deviation; FTND = Fagerström Test for Nicotine Dependence

### Feasibility outcomes

During the recruitment period, 22 psychiatric outpatients were screened for eligibility. Of these, 18 met inclusion criteria and consented to participate (recruitment rate: 81.8%).

Sixteen participants attended at least 75% of scheduled sessions (session adherence: 88.9%), and 15 completed the full 12-week program (retention rate: 83.3%).

All follow-up visits were conducted as scheduled without disruption to existing psychiatric services. Integration of the cessation clinic required minor scheduling adjustments but was successfully incorporated into routine outpatient care. See Table 2.

**Table 2:** Feasibility and acceptability indicators (*N* = 18)

Indicator	Definition	Criteria	Result
1. Recruitment rate	% of screened patients enrolled	≥ 80%	81.8% (18/22)
2. Participation rate	% attending ≥ 75% of sessions	≥ 80%	88.9% (16/18)
3. Retention rate	% completing 12-week program	≥ 80%	83.3% (15/18)
4. Patient satisfaction (mean ± SD)	Self-report (5-point scale)	≥ 4.0	4.47 ± 0.38
5. Staff satisfaction (mean ± SD)	Self-report (5-point scale)	≥ 4.0	4.52 ± 0.22

Scores collected using 5-point Likert scale (1 = very dissatisfied, 5 = very satisfied)

### Acceptability outcomes

Mean patient satisfaction score was 4.60 ± 0.50 on a 5-point Likert scale. Staff members involved in the program reported a mean satisfaction score of 4.3 out of 5.0.

Qualitative feedback indicated that participants valued the convenience of receiving smoking cessation support within routine psychiatric care, the accessibility of the pharmacist, and the supportive, nonjudgmental clinical environment. Service users also emphasized the benefit of continuity between smoking cessation counseling and ongoing psychiatric follow-up.

### Preliminary service outcomes

At Week 12, 7-day point prevalence abstinence rate (intention-to-treat) was 44.4% (8/18), and 30-day point prevalence abstinence was 27.8% (5/18). The Mean daily cigarette consumption significantly decreased from 18.3 ± 4.2 at baseline to 7.9 ± 3.7 in Week 12 (*p* < 0.01), while exhaled carbon monoxide levels declined from 14.2 ppm to 5.6 ppm (*p* < 0.01). Motivation to quit improved significantly, increasing from 5.1 to 7.8 (*p* < 0.01), and the psychological quality of life increased significantly from 46.2 points at baseline to 61.4 points at Week 12 (*p* < 0.01). No serious adverse drug reactions or psychiatric relapses were observed during the 12-week follow-up period. See Table 3.

**Table 3:** Preliminary service outcomes after 12 weeks (*N* = 18)

Outcome Variable	Baseline (Mean ± SD)	Week 12 (Mean ± SD)	*p*-value	Effect Size (Hedges’ g)
1. 7-day abstinence (n, %)	0 (0%)	8 (44.4%)	–	–
2. 30-day abstinence (n, %)	0 (0%)	5 (27.8%)	–	–
3. Cigarettes/day	18.3 ± 4.2	7.9 ± 3.7	< 0.01	2.51
4. CO (ppm)	14.2 ± 6.9	5.6 ± 3.2	< 0.01	1.53
5. Motivation to Quit (0–10)	5.1 ± 1.3	7.8 ± 1.1	< 0.01	2.15
6. WHOQOL-BREF (Psychological)	46.2 ± 9.5	61.4 ± 7.8	< 0.01	1.67

*p*-values from paired t-tests; CO = carbon monoxide; WHOQOL-BREF = World Health Organization Quality of Life - Brief Version; SD = Standard Deviation

### Implementation observations

The collaborative care model functioned through structured coordination between the clinical pharmacist, psychiatrists, and psychiatric nurses. The pharmacist served as the primary coordinator for medication management, behavioral counseling, and motivational interviewing, while psychiatrists oversaw psychiatric treatment adjustments as needed.

Operational facilitators included institutional leadership support, pharmacist training in motivational interviewing, and flexible scheduling arrangements for the pilot clinic. Reported operational challenges included staffing constraints during off-hour clinic sessions and occasional scheduling overlap with routine psychiatric medication appointments.

## Discussion

This pilot study provides empirical insights into the feasibility, acceptability, and early service-level outcomes of a pharmacist-led holistic smoking cessation clinic embedded within psychiatric care services in a provincial Thai hospital. The initiative was grounded in quality improvement and patient-centered service innovation rather than experimental hypothesis testing.

The findings indicate that integration of a pharmacist-led cessation model within routine psychiatric care is operationally feasible and acceptable to both patients and staff. Recruitment, session adherence, and retention rates exceeded 80%, which is notable given the known logistical challenges, stigma, and motivational barriers associated with smoking cessation among individuals with mental illness [3, 4]. The relatively high engagement observed in this pilot may reflect the advantage of embedding cessation services within established therapeutic relationships and familiar clinical environments.

The 7-day point prevalence abstinence rate of 44.4% in Week 12 suggests potential short-term effectiveness, although interpretation should remain cautious given the pilot design and absence of a control group. These findings are broadly consistent with prior reports demonstrating that integrated pharmacologic and behavioral strategies can improve cessation outcomes among psychiatric populations [12]. The use of multiple pharmacological options—including cytisine and culturally familiar herbal support such as Cyanthillium cinereum —alongside brief behavioral counseling and family engagement reflects a pragmatic, locally adapted approach. *Cyanthillium cinereum*, or herbal tea, has been recognized as one of the most popular herbal smoking cessation aids used widely [5]. Contemporary global tobacco control frameworks emphasize the importance of tailoring cessation interventions to cultural and health system contexts to enhance engagement and adherence [17].

From an implementation perspective, several operational elements appeared to support the model. Institutional endorsement and designation as a service development initiative facilitated alignment with hospital workflows. Pharmacist training in motivational interviewing expanded the scope of patient engagement beyond medication dispensing, consistent with the evolving role of pharmacists in behavioral health care [4]. Weekly interdisciplinary case discussions supported coordination of psychiatric medication adjustments and follow-up scheduling within routine services.

In addition to smoking-related indicators, improvements were observed in motivation to quit and psychological quality of life scores. While these outcomes were self-reported and should be interpreted cautiously, they are consistent with literature suggesting that smoking cessation may be associated with improvements in mental health over time [15]. Staff satisfaction with the integrated model further suggests that embedding cessation care within psychiatric services may be acceptable from a provider perspective.

Nevertheless, the primary objective of this initiative was to examine the practicality of embedding a pharmacist-led cessation clinic within a real-world, resource-constrained psychiatric hospital. In this regard, the findings align with broader calls for implementation-oriented research and adaptive service development in mental health systems [6]. By prioritizing feasibility, contextual adaptation, and integration within existing workflows, this pilot contributes to emerging evidence on scalable tobacco cessation strategies for vulnerable populations.

Future work should include longer-term follow-up, refinement of referral pathways, and structured training frameworks for clinical pharmacists. Expansion through digital support tools, such as SMS reminders or teleconsultation, may further enhance adherence and continuity of care. A larger controlled evaluation would be required to determine long-term effectiveness and cost-efficiency before wider scale-up.

### Strengths and limitations

This pilot study represents an early effort to integrate a pharmacist-led smoking cessation model within routine psychiatric outpatient care in Thailand. The service was developed through stakeholder-informed co-design and incorporated both objective measures (e.g., exhaled CO monitoring) and validated instruments (FTND, Motivation to Quit Scale, WHOQOL-BREF Thai version), supporting the methodological of the findings.

Several limitations warrant consideration. The sample size was small and based on convenience recruitment, limiting generalizability. The absence of a comparison group restricts causal inference. Self-reported measures—including motivation and quality of life—may be influenced by social desirability bias. Furthermore, the 12-week follow-up period does not capture long-term abstinence or relapse trajectories, which are particularly relevant in psychiatric populations characterized by fluctuating symptoms and medication changes.

### Future directions

Building on the findings of this pilot, future research should evaluate the model in larger and more diverse psychiatric settings. A controlled or quasi-experimental design with extended follow-up would allow more robust assessment of clinical effectiveness, relapse prevention, and long-term quality-of-life outcomes.

Further refinement of referral pathways, structured training in motivational interviewing, and standardized clinical workflows may enhance consistency and scalability of the service. Development of formal training modules for clinical pharmacists could support workforce capacity in integrated cessation care.

In addition, integration of digital follow-up tools—such as SMS reminders or teleconsultation—may improve adherence and accessibility, particularly in rural settings. Broader collaboration with primary care and community services would also support continuity of care beyond the hospital setting.

## Conclusion

This pilot study suggests that a pharmacist-led holistic smoking cessation clinic can be integrated within routine psychiatric outpatient care in a Thai mental health facility. The multidisciplinary model was operationally feasible and acceptable to both patients and staff, with encouraging short-term smoking cessation outcomes.

Although limited by small sample size and short follow-up duration, the findings provide preliminary support for further evaluation of pharmacist-led smoking cessation services in psychiatric settings. Future controlled studies with longer follow-up are warranted to determine long-term effectiveness and scalability.

## Data Availability

The datasets generated and analyzed during this study are not publicly available due to institutional policies and the sensitive nature of psychiatric clinical data. De-identified data may be available from the corresponding author upon reasonable request and subject to institutional approval.
